# Cellular inorganic carbon fluxes in *Trichodesmium*: a combined approach using measurements and modelling

**DOI:** 10.1093/jxb/eru427

**Published:** 2014-11-26

**Authors:** Meri Eichner, Silke Thoms, Sven A. Kranz, Björn Rost

**Affiliations:** ^1^Marine Biogeosciences, Alfred Wegener Institute, Helmholtz Centre for Polar and Marine Research, Am Handelshafen 12, 27570 Bremerhaven, Germany; ^2^Department for Geosciences, Princeton University, Princeton, NJ 08540, USA; ^3^ Present address: Department of Earth, Ocean and Atmospheric Sciences, Florida State University, Tallahassee, Fl 32306, USA

**Keywords:** Carbon acquisition, carbon-concentrating mechanism (CCM), CO_2_, cyanobacteria, leakage, NDH, ocean acidification.

## Abstract

To predict effects of climate change on phytoplankton, it is crucial to understand how their mechanisms for carbon acquisition respond to environmental conditions. Aiming to shed light on the responses of extra- and intracellular inorganic C (C_i_) fluxes, the cyanobacterium *Trichodesmium erythraeum* IMS101 was grown with different nitrogen sources (N_2_ vs NO_3_^–^) and *p*CO_2_ levels (380 vs 1400 µatm). Cellular C_i_ fluxes were assessed by combining membrane inlet mass spectrometry (MIMS), ^13^C fractionation measurements, and modelling. Aside from a significant decrease in C_i_ affinity at elevated *p*CO_2_ and changes in CO_2_ efflux with different N sources, extracellular C_i_ fluxes estimated by MIMS were largely unaffected by the treatments. ^13^C fractionation during biomass production, however, increased with *p*CO_2_, irrespective of the N source. Strong discrepancies were observed in CO_2_ leakage estimates obtained by MIMS and a ^13^C-based approach, which further increased under elevated *p*CO_2_. These offsets could be explained by applying a model that comprises extracellular CO_2_ and HCO_3_^–^ fluxes as well as internal C_i_ cycling around the carboxysome via the CO_2_ uptake facilitator NDH-1_4_. Assuming unidirectional, kinetic fractionation between CO_2_ and HCO_3_^–^ in the cytosol or enzymatic fractionation by NDH-1_4_, both significantly improved the comparability of leakage estimates. Our results highlight the importance of internal C_i_ cycling for ^13^C composition as well as cellular energy budgets of *Trichodesmium*, which ought to be considered in process studies on climate change effects.

## Highlight text

Combining experimental and theoretical approaches to characterize the carbon acquisition of a cyanobacterium, this paper improves interpretations of C isotope fractionation by accounting for the important role of intracellular C cycling.

## Introduction

Cyanobacteria are ancient organisms responsible for oxygenation of the atmosphere during times when CO_2_ concentrations were about two orders of magnitude higher than today (cf. Buick, 1992; Kasting and Siefert, 2002). Possibly due to their origin at that time, the CO_2_-fixing enzyme RubisCO of cyanobacteria has one of the lowest affinities among all autotrophic organisms (Badger *et al.*, 1998; Tortell, 2000). Consequently, cyanobacteria are dependent on high activities of carbon-concentrating mechanisms (CCM) for increasing the CO_2_ concentration in the vicinity of RubisCO. Currently, due to ongoing anthropogenic CO_2_ combustion, the availability and speciation of inorganic C (C_i_) in seawater is changing at a rapid pace (IPCC, 2007). In view of this ocean acidification (Caldeira and Wickett, 2003), a number of studies in recent years have focused on the mechanisms of C acquisition and CO_2_ responses of different groups of phytoplankton (e.g. Rost *et al.*, 2008). Among these studies, the abundant N_2_-fixing cyanobacterium *Trichodesmium* stands out by showing an exceptionally high stimulation of biomass production and N_2_ fixation in response to elevated *p*CO_2_ (e.g. Hutchins *et al.*, 2007; Levitan *et al.*, 2007; Kranz *et al.*, 2009). Further studies on the underlying reasons for these CO_2_ effects show a decrease in C_i_ affinity at high *p*CO_2_ (Kranz *et al.*, 2009; Kranz *et al.*, 2010). Given the high energy demand of the CCM in cyanobacteria, a reallocation of energy between C_i_ acquisition and N_2_ fixation was suggested to stimulate production at high *p*CO_2_ (Kranz *et al.*, 2010).

Cellular C_i_ affinities of *Trichodesmium* are determined by the interplay of several transporters and structural adaptations composing the CCM. In order to understand *p*CO_2_ responses of the CCM as well as potential changes in energy demand, it is necessary to distinguish between these different components. While CO_2_ can diffuse through the cell membrane without energy investments, the low equilibrium concentrations, slow interconversion with HCO_3_^–^ (Zeebe and Wolf-Gladrow, 2007), and its tendency to leak out of the cell compromise the use of CO_2_ as the predominant C_i_ source. Therefore, cyanobacteria have evolved energy-dependent transporters for taking up HCO_3_^–^, which can be accumulated in the cell more efficiently (Badger *et al.*, 2006). *Trichodesmium* has been found to cover ~90% of its C demand using HCO_3_^–^ (Kranz *et al.*, 2009; Kranz *et al.*, 2010). Uptake of HCO_3_^–^ in this species is catalysed by the Na^+^-dependent transporter BicA, which is fuelled by Na^+^/HCO_3_^–^ symport or via an H^+^/Na^+^ antiport mechanism (Price *et al.*, 2008).

Cyanobacterial RubisCO is localized in distinct compartments within the cell, the so-called carboxysomes. The protein shells of these microbodies are permeable to HCO_3_^–^ but pose a diffusion barrier for CO_2_ (Dou *et al.*, 2008; Espie and Kimber, 2011), allowing significant accumulation of CO_2_ in the vicinity of RubisCO. Inside the carboxysomes, transformation of HCO_3_^–^ to CO_2_ is accelerated by carbonic anhydrase (CA; reviewed by Espie and Kimber, 2011). In addition to direct HCO_3_^–^ uptake and CO_2_ diffusion, CO_2_ uptake in *Trichodesmium* is facilitated by the NDH-1_4_ complex, which converts CO_2_ to HCO_3_^–^ in the cytoplasm, presumably in a CA-like reaction (Price *et al.*, 2002). The protein complex is thought to be located on the thylakoid membrane and form part of the photosynthetic/respiratory electron transport chain, being fuelled by electrons donated from NADPH or ferredoxin, which are subsequently transferred to the plastoquinone pool (Price *et al.*, 2002). After the hydration of CO_2_, a proton is thought to be released into the thylakoid lumen, contributing to the pH gradient necessary for ATP synthesis and making the reaction irreversible in the light (Price *et al.*, 2002).

Conversion of CO_2_ to HCO_3_^–^ by the NDH complex has been proposed to drive internal C_i_ recycling to minimize loss via CO_2_ efflux (Maeda *et al.*, 2002; Price *et al.*, 2002). Due to the strong CO_2_ accumulation required in cyanobacteria, CO_2_ efflux is a major challenge in these organisms. Despite the interplay of the carboxysome and proposed recapture of CO_2_ by the NDH-1_4_ complex, efflux of CO_2_ has been shown to equal ~50–90% of gross C_i_ uptake in *Trichodesmium* (Kranz *et al.*, 2009; Kranz *et al.*, 2010). Next to the C source (CO_2_ vs HCO_3_^–^), leakage (i.e. CO_2_ efflux : gross C_i_ uptake) can strongly affect isotopic composition of organic C produced during photosynthesis (Burkhardt *et al.* 1999, Sharkey and Berry, 1985), and thus measurements of ^13^C fractionation can provide complementary information on this aspect of CCM regulation (e.g. Laws *et al.*, 1997; Keller and Morel, 1999; Rost *et al.*, 2006; Tchernov and Lipschultz, 2008). In fact, differences in leakage estimates based on membrane inlet mass spectrometry (MIMS; Badger *et al.*, 1994) and C isotope fractionation (Sharkey and Berry, 1985) have been attributed to internal C_i_ cycling driven by NDH (Kranz *et al.*, 2010).

In a previous study (Eichner *et al.*, 2014), the energy allocation to different physiological processes in *Trichodesmium* under varying energetic states was addressed by altering the cellular energy budget through addition of different nitrogen sources: while N_2_ fixation is a highly energy-demanding process with a high demand for ATP, NO_3_^–^ requires very little ATP (only for uptake) but instead has a high electron demand. The study highlighted the dependence of energy reallocation on the stoichiometry in energy demands (ATP vs NADPH) of the different pathways involved. The energy demand of the CCM in *Trichodesmium* remains uncertain, however, especially because the regulation of internal C_i_ fluxes is as yet poorly characterized. To shed light on the extra- and intracellular C_i_ fluxes under the different energetic conditions, *Trichodesmium* was grown with different *p*CO_2_ levels and N sources (N_2_ vs NO_3_^–^), and a combination of different methods, including MIMS and ^13^C fractionation measurements, as well as modelling, was employed. While MIMS provides a useful tool to investigate C_i_ fluxes across the cell membrane, internal fluxes cannot be directly measured and were therefore modelled. Model calculations of internal C_i_ fluxes made use of the measured extracellular C_i_ fluxes and the isotopic composition of particulate organic C (δ^13^C_POC_), which reflects the integrated effects of extra- and intracellular C_i_ fluxes. Hereby, a common model of ^13^C fractionation (Sharkey and Berry, 1985) was extended by including internal fluxes around the carboxysome.

## Materials and methods

### Culture conditions

*Trichodesmium erythraeum* IMS101 was grown in semi-continuous batch cultures at 25°C and 150 μmol photons m^–2^ s^–1^ with a 12 h : 12h light : dark cycle. Cultures were grown in 0.2-μm-filtered artificial seawater (YBCII medium; Chen *et al.*, 1996) and kept in exponential growth phase by regular dilution with culture medium. Culture bottles were continuously bubbled with 0.2-μm-filtered air with *p*CO_2_ levels of 380 and 1400 μatm. Prior to experiments, cells were allowed to acclimate to the respective *p*CO_2_ for at least 2 weeks. Cultures in which pH had drifted by >0.09 units compared to cell-free reference media were excluded from further analysis. In treatments with NO_3_^–^ as the N source, 0.2-μm-filtered NaNO_3_ was added to achieve mean concentrations of 97±2 µmol l^–1^ in the experiments, and these never fell below 65 µmol l^–1^. Cultures were acclimated to NO_3_^–^ for at least 1 week before measurements. Samples for the analysis of dissolved inorganic C (DIC) were filtered through 0.2 μm filters and measured colourimetrically (QuAAtro autoanalyzer, Seal, Norderstedt, Germany). Average precision was ±5 μmol kg^–1^. The pH values of the acclimation media were measured potentiometrically (pH meter pH3110, WTW, Weilheim, Germany). For further details on culture conditions as well as carbonate chemistry parameters, see Eichner *et al.* (2014).

### MIMS measurements

**Fig. 1.**
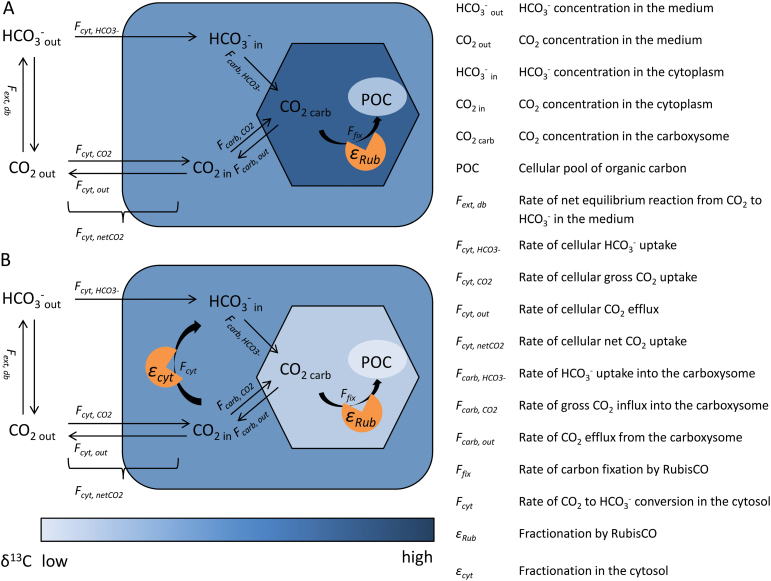
Schematic diagram showing the cellular C_i_ pools and fluxes characterized by measurements and modelling. Fluxes and concentrations in the external medium and fluxes over the cell membrane as well as C fixation (*F*_*fix*_) were measured by MIMS, while fluxes in and out of the carboxysome were modelled. Shading intensity denotes δ^13^C values of different cellular C pools (including POC and C_i_ in the cytosol and carboxysome). (A) Fractionation during C fixation by RubisCO leads to depletion of POC in ^13^C and enrichment of ^13^C in the carboxysomal C_i_ pool. (B) Fractionation during internal C_i_ cycling, e.g. via NDH, leads to ^13^C depletion of the carboxysomal C_i_ pool. Consequently, the POC formed is isotopically lighter than in scenario A. This figure is available in colour at *JXB* online.

Cellular C_i_ fluxes (Fig. 1) were obtained using a custom-made MIMS system (Rost *et al.*, 2007), applying a disequilibrium approach described by Badger *et al.* (1994). Assays were performed in YBCII medium buffered with HEPES (50mM, pH 8.0) at acclimation temperature and light intensity, unless otherwise specified. To account for the diurnal cycle of C_i_ fluxes in *Trichodesmium*, measurements were performed three times over the day, during time intervals from 0 to 1.5, 5.5 to 7, and 9 to 10.5h after beginning of the photoperiod. CO_2_ and O_2_ fluxes were measured as a function of DIC, starting with concentrations close to zero (media bubbled with CO_2_-free air), which were subsequently increased by step-wise addition of NaHCO_3_ up to concentrations of ~5000 μM. As the assay medium is buffered, unlike the conditions during acclimation of the cells, the HCO_3_^–^:CO_2_ ratio stayed constant over the investigated DIC range. DIC-saturated rates of photosynthesis (V_max_) and half-saturation concentrations [K_1/2_ (DIC)] were obtained by fitting a Michaelis-Menten function to the data. Net O_2_ evolution was converted to C fixation (*F*_*fix*_) assuming a photosynthetic quotient (PQ) of 1.34 (Williams and Robertson, 1991). Net CO_2_ uptake (*F*_*cyt, netCO2*_) was calculated from the steady-state rate of CO_2_ depletion at the end of the light period and corrected for the CO_2_/HCO_3_^–^ interconversion in the medium (*F*_*ext, db*_). Using C fixation and net CO_2_ uptake, HCO_3_^–^ uptake rates (*F*_*cyt, HCO3–*_) could be derived by a mass balance equation:

$$F_{cyt,HCO_{3}^{-}}=F_{fix}-F_{cyt,netCO_{2}}$$ (1)

For normalization of the CO_2_ and O_2_ traces, duplicate samples for chlorophyll *a* (chl *a*) analysis were taken after each measurement. Chl *a* was extracted in acetone for >12h and determined fluorometrically (TD-700 fluorometer, Turner Designs, Sunnyvale, CA, USA; Holm-Hansen and Rieman, 1978).

### Leakage estimation

Cellular leakage was estimated by two different methods. Firstly, leakage was determined by MIMS measurements using the disequilibrium approach (Badger *et al.*, 1994). Cellular leakage (*L*_*MIMS*_) is defined as the ratio of CO_2_ efflux (*F*_*cyt, out*_) to gross C_i_ uptake [i.e. the sum of HCO_3_^–^ (*F*_*cyt, HCO3–*_) and gross CO_2_ uptake (*F*_*cyt, CO2*_)]:

$$L_{MIMS}=\frac{F_{cyt,\;out}}{F_{cyt,\;HCO_{3}^{-}}+F_{cyt,\;CO_{2}^{}}}$$ (2)

*F*_*cyt, out*_ was estimated from the initial increase in CO_2_ concentration after switching off the light (Badger *et al.*, 1994). These estimates are based on the assumption that the rate of diffusive CO_2_ efflux during the light phase is well represented by the rate of CO_2_ efflux during the first ~20 s of the subsequent dark phase. As leakage calculated by this approach is based on O_2_ measurements that are converted to C fluxes, the sensitivity to different PQ values was tested by varying PQ between 1.0 and 1.7, yielding deviations of not more than 15% of leakage estimates (i.e. 0.06 units).

In the second approach, leakage was estimated from the isotopic fractionation during POC formation (*ε*_*p*_), which was calculated from the difference in isotopic composition between POC (δ^13^C_POC_) and CO_2_ (δ^13^C_CO2_) in the medium according to Freeman and Hayes (1992). Duplicate samples for analysis of δ^13^C_POC_ were filtered onto pre-combusted GF/F filters and acidified with 200 μl HCL (0.2M) to remove all C_i_ prior to analysis. δ^13^C_POC_ was measured with an EA mass spectrometer (ANCA SL 2020, SerCon Ltd, Crewe, UK). For analysis of the isotopic composition of DIC (δ^13^C_DIC_), filtered samples were fixed with HgCl_2_ (final concentration 110mg l^–1^). Subsequent to acidification of the samples, isotopic composition of CO_2_ in the headspace was analysed with an isotope ratio mass spectrometer (GasBench-II coupled to Delta-V advantage, Thermo, Bremen, Germany). The isotopic composition of CO_2_ was calculated from δ^13^C_DIC_, following a mass balance equation (Zeebe and Wolf-Gladrow, 2007). Leakage (*L*_*13C*_) was subsequently derived using an extended equation from Sharkey and Berry (1985):

$$L_{13C}=\frac{\varepsilon_{p}-(a_{cyt}\varepsilon_{db})}{\varepsilon_{Rub}}$$ (3)

where *ε*_*Rub*_ is the intrinsic discrimination of ^13^C by RubisCO (assumed to be +25‰; Roeske and O’Leary, 1984; Guy *et al.*, 1993) and *ε*_*db*_ represents the equilibrium fractionation between CO_2_ and HCO_3_^–^ (–9‰; Mook *et al.*, 1974). The fractional contribution of HCO_3_^–^ to gross C_i_ uptake (*a*_*cyt*_), being introduced by Burkhardt *et al.* (1999), has been determined by MIMS measurements for the respective treatments. These calculations assume an equilibrium situation and further consider the cell as a single compartment.

## Results and discussion

### General CCM characteristics

**Fig. 2.**
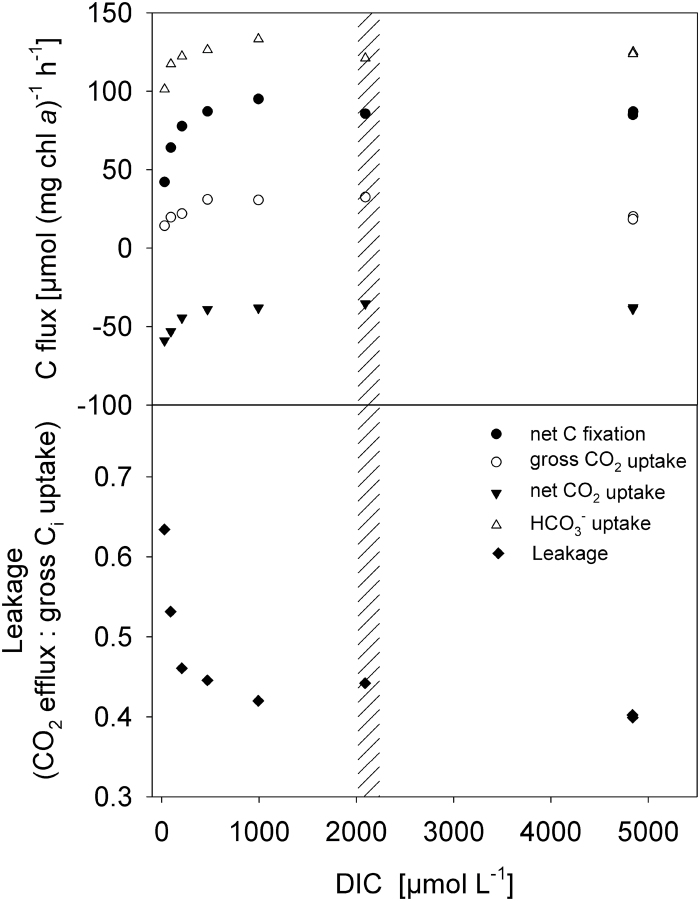
Example showing the dependence of C_i_ fluxes measured by MIMS in *Trichodesmium* on the DIC concentration in the assay. Data shown were measured in the evening in a culture grown at 380 µatm *p*CO_2_ without NO_3_^–^. The shaded area denotes the range of acclimation DIC levels.

MIMS measurements showed a highly efficient CCM with a high capacity for regulation of C_i_ affinity over the diurnal cycle as well as with different *p*CO_2_ levels, in agreement with previous studies on *Trichodesmium* (e.g. Kranz *et al.*, 2009; Kranz *et al.*, 2010). Half-saturation DIC concentrations for C fixation (K_1/2_) ranged between ~20 and 500 µmol DIC l^–1^ (Supplementary Figure S1), which is equivalent to ~0.2 and 4 µmol CO_2_ l^–1^ and is thus substantially lower than the K_M_ of cyanobacterial RubisCO (105–185 µmol CO_2_ l^–1^; Badger *et al.*, 1998). Taking the ratio of K_M_ to K_1/2_ as a measure of CO_2_ accumulation in the vicinity of RubisCO (assuming a K_M_ of 150 µmol CO_2_ l^–1^), our data suggest accumulation factors between ~35 and 900 and indicate that the degree of RubisCO saturation is always larger than 80%. Accordingly, under the applied external CO_2_ concentrations, concentrations in the carboxysome typically exceed 600 µmol CO_2_ l^–1^. The CCM was primarily based on active HCO_3_^–^ uptake, accounting for 82±4% of gross C_i_ uptake (Table 1). As gross C_i_ uptake was approximately twice as high as net C fixation at acclimation DIC (~2100 µmol CO_2_ l^–1^), leakage measured by MIMS ranged between 0.3 and 0.7 (i.e. CO_2_ efflux equalled 30–70% of gross C_i_ uptake; Table 1). As a consequence of the high HCO_3_^–^ contribution and the high CO_2_ efflux, the net fluxes of CO_2_ were generally directed out of the cell (cf. negative values for net CO_2_ uptake: Table 1, Fig. 2).

**Table 1.** Diurnal cycle of C_i_ fluxes measured by MIMS under acclimation DIC levels (~2100 µmol l^–1^) in *Trichodesmium* acclimated to two different *p*CO_2_ levels (380 vs 1400 µatm) and N sources (N_2_ vs NO_3_^–^)^a^

	380 µatm –NO_3_^–^			380 µatm +NO_3_^–^			1400 µatm –NO_3_^–^			1400 µatm +NO_3_^–^		
	Morning	Midday	Evening	Morning	Midday	Evening	Morning	Midday	Evening	Morning	Midday	Evening
Net C fixation	91±15	57±14	87±3	95±20	56±22	70±18	80±13	39±14	61±4	91	61±19	63±16
Gross C_i_ uptake	157±18	144±24	167±17	135±23	128±16	143±25	144±7	124±9	142±6	144	127±22	131±17
HCO_3_^–^ uptake	134±18	117±22	133±12	118±21	108±14	113±14	126±11	95±9	112±2	120	97±17	102±12
Gross CO_2_ uptake	23±4	27±4	34±6	17±3	20±2	30±13	18±4	28±5	30±6	24	30±9	29±6
Net CO_2_ uptake	–45±10	–60±12	–46±9	–23±2	–52±8	–42±11	–46±5	–56±8	–51±4	–28	–36±8	–39±9
HCO_3_^–^:C_i_ uptake	0.85±0.03	0.81±0.03	0.80±0.02	0.87±0.01	0.84±0.01	0.80±0.06	0.88±0.03	0.77±0.04	0.79±0.03	0.83	0.76±0.05	0.78±0.01
CO_2_ efflux	69±12	87±13	79±13	40±3	72±6	72±8	64±7	85±14	81±3	53	66±4	67±10
Leakage	0.44±0.05	0.61±0.05	0.47±0.03	0.30±0.03	0.57±0.11	0.51±0.04	0.45±0.07	0.69±0.10	0.57±0.01	0.37	0.53±0.07	0.52±0.07

^a^ All C_i_ fluxes are given in µmol C (mg chl *a*)^–1^ h^–1^. Errors are standard deviations for biological replicates (1 SD; *n* = 3 except 1400 +NO_3_^–^ morning with *n* = 1).

#### Diurnal changes in C_i_ fluxes

The diurnal cycle was characterized by low K_1/2_ values in the morning and a downregulation of C fixation rates at midday (ANOVA, *P* < 0.001; Supplementary Figure S1A and B). Leakage estimated by MIMS at acclimation DIC was lowest in the morning, increased towards midday, and decreased again towards the evening (ANOVA, *P* < 0.05; Table 1). Leakage estimates for DIC levels approaching zero (obtained by curve fits of leakage plotted over DIC concentration; Fig. 2) varied even more over the course of the day, yielding values around 0.3 in the mornings, while at midday and in the evening ratios approached 1.0 (data not shown). These diurnal changes in leakage could be explained by the concurrent changes in the ratio of HCO_3_^–^ to CO_2_ uptake (Table 1, Supplementary Figure 1C), which were characterized by low CO_2_ fluxes in the mornings (ANOVA, *P* < 0.05; Table 1), while HCO_3_^–^ uptake was higher in the morning than at midday, and increased again towards the evening (ANOVA, *P* < 0.05; Table 1). Over the day, a higher share of HCO_3_^–^ uptake, which is less prone to diffuse out of the cell, was thus correlated with lower leakage.

#### Effects of different pCO_2_ levels and N sources

The affinity for C_i_ was downregulated at elevated *p*CO_2_, as indicated by high K_1/2_ values under these conditions (Supplementary Figure 1B). Under acclimation DIC, however, C_i_ fluxes (C fixation, C_i_ uptake, and CO_2_ uptake and efflux) were not significantly affected by *p*CO_2_ (ANOVA, *P* > 0.05; Table 1), reflecting the cells’ capacity to achieve similar C fixation over a range of *p*CO_2_ levels by regulating their CCM. Regarding the N source, C fixation rates and CO_2_ uptake under acclimation DIC were equally unaffected (ANOVA, *P* > 0.05; Table 1). Although cells mainly used HCO_3_^–^ as a C_i_ source in all treatments, HCO_3_^–^ uptake at acclimation DIC decreased slightly with increasing *p*CO_2_ (~10%; ANOVA, *P* < 0.05; Table 1, Supplementary Figure 1C), but was not affected by N source (ANOVA, *P* > 0.05; Table 1). Interestingly, CO_2_ efflux was affected by the N source (ANOVA, *P* < 0.01; Table 1), with ~20% lower efflux in NO_3_^–^ users compared to N_2_ fixers, possibly due to differences in internal pH caused by the uptake/accumulation of NO_3_^–^ vs NH_4_^+^ in the cell. One could also speculate that growing cells on NO_3_^–^ reduces the general membrane permeability, since NH_4_^+^ transfer between cells is only necessary under N_2_-fixing conditions, which could also affect the permeability for CO_2_. Leakage at acclimation DIC estimated by MIMS was, however, not significantly affected by *p*CO_2_ or N source at any time of the day (ANOVA, *P* > 0.05; Table 1).

### Offsets in leakage estimates

**Fig. 3.**
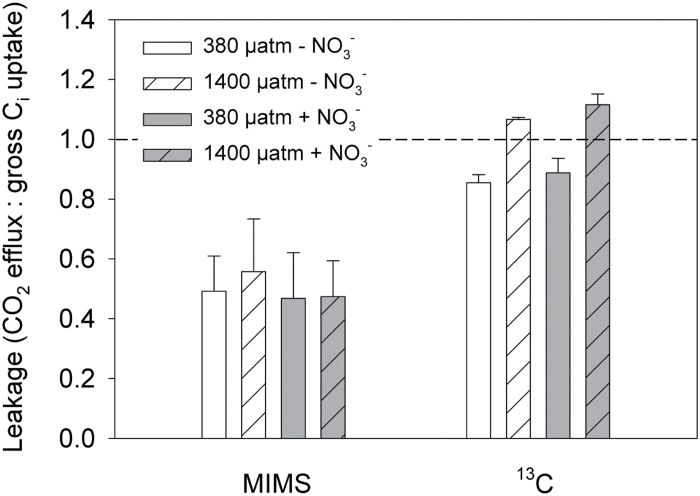
Leakage estimates by MIMS (mean values of measurements conducted at three time points over the day; Badger *et al.*, 1994) and ^13^C fractionation (Sharkey and Berry, 1985) determined in *Trichodesmium* grown under two *p*CO_2_ levels and N sources (*n* ≥ 3).

High leakage values obtained in MIMS measurements reflect the strong C_i_ accumulation necessary for C fixation in cyanobacteria due to the poor CO_2_ affinity of their RubisCO. However, leakage estimates obtained from δ^13^C values (*L*_*13C*_, eqn 3) even exceeded MIMS-based estimates. Overall fractionation during formation of POC (*ε*_*p*_) was not significantly affected by N treatment (ANOVA, *P* > 0.05) but increased with *p*CO_2_ (ANOVA, *P* < 0.0001), ranging from 14.4±1.0‰ at 380 µatm to 19.9±0.9‰ at 1400 µatm *p*CO_2_. Consequently, leakage estimates based on *ε*_*p*_ (eqn 3) also increased with *p*CO_2_, while estimates from MIMS measurements at acclimation DIC were constant over the range of *p*CO_2_ levels. *L*_*13C*_ was calculated to range between 0.82 and 1.14, exceeding MIMS-based measurements by ~30–60% (Fig. 3) and even reaching theoretically impossible values (>1). A similar discrepancy between these two approaches, which was equally dependent on *p*CO_2_ acclimation, has been observed previously (Kranz *et al.*, 2010). In the following paragraph, possible reasons for the deviations between estimates are outlined.

Following the approach by Badger *et al.* (1994), leakage is directly calculated from the measured CO_2_ efflux and gross C_i_ uptake. As CO_2_ efflux cannot readily be determined during the light due to the concurrent C_i_ uptake, the rise in the CO_2_ signal directly after switching off the light is taken as an estimate of CO_2_ efflux during the light phase, assuming that the accumulated C_i_ pool and therewith gross CO_2_ efflux are initially at the pre-darkness level (Badger *et al.*, 1994). If active C_i_ uptake as well as C fixation by RubisCO do not cease immediately upon darkening, leakage estimates could be biased and likely to be underestimated. Despite these potential uncertainties, this is a more direct approach than the alternative method, which infers leakage from the isotopic composition of cells. The ^13^C-based approach makes use of the effect of leakage on *ε*_*p*_ (eqn 3; Sharkey and Berry, 1985). Briefly, while the intrinsic fractionation by RubisCO (*ε*_*Rub*_) generally causes organic material to be depleted in ^13^C (Fig. 1A), variation in *ε*_*p*_ can be induced by changes in the C_i_ source and/or leakage. Consequently, any errors in estimates of *ε*_*Rub*_ or *a*_*cyt*_, but also any unaccounted process affecting *ε*_*p*,_ would cause ^13^C-based leakage estimates to be biased.

Kranz *et al.* (2010) suggested that internal C_i_ cycling within the cell may affect *ε*_*p*_ in general. The CO_2_ dependence of the offset between MIMS- and ^13^C-based leakage estimates was furthermore suggested to reflect a CO_2_ effect on the NDH complex driving this internal C_i_ cycling (Kranz *et al.*, 2010), in line with early observations of the C_i_ dependence of CO_2_ uptake by the NDH complex (Price and Badger, 1989a,c). In Fig. 1, the effects of internal C_i_ cycling on isotopic composition are illustrated. While Fig. 1A assumes an equilibrium situation and does not include any internal C_i_ cycling, Fig. 1B illustrates non-equilibrium situations caused by internal C_i_ cycling. The degree of ^13^C enrichment in the cytosol and within the carboxysome, according to this concept, would be dependent on the type of kinetic fractionation in the cytosol. This could include complete or incomplete unidirectional fractionation as well as enzymatic fractionation by the NDH complex. Accounting for these processes requires the introduction of a second compartment. The approach taken here can be considered as an extension of the model of Sharkey and Berry (1985), which considers the cell as one compartment. In order to avoid errors being introduced by large uncertainties, e.g. in permeability of the plasma membrane and carboxysome in *Trichodesmium*, a flux-based model that is independent of these assumptions is employed, rather than a full kinetic model. Our approach is similar to the model of Schulz *et al.* (2007), but disequilibrium situations are also considered.

### Internal C_i_ fluxes and fractionation—model setup

To test our concept (Fig. 1) and quantitatively describe the possible effect of internal cycling on δ^13^C, intracellular C_i_ fluxes and their effects on isotopic ratios in different cellular C_i_ pools were modelled. For parameterizations, HCO_3_^–^ and gross CO_2_ fluxes measured by MIMS as well as measured fractionation values *ε*_*p*_ were used. The model is based on flux balance equations for the individual isotope species. The flux balance of total C (^12^C + ^13^C) in the cytosol and in the carboxysome, respectively, is given by the following equations:

$$\begin{array}{l} F_{cyt,\;CO_{2}}+F_{cyt,\;HCO_{3}^{-}}+F_{carb,\;out}\;\\ -\;F_{cyt,\;out}-F_{carb,\;CO_{2}}-F_{carb,\;HCO_{3}^{-}}=0 \end{array}$$ (4)

$$F_{carb,\;HCO_{3}^{-}}+F_{carb,\;CO_{2}}-F_{carb,\;out}-F_{fix}=0$$ (5)

As about 99% of C is ^12^C, i.e. F=F12+F13≡F12, the flux balance equations for ^13^C can be derived by multiplying the fluxes (*F*) with the isotopic ratio *R* = ^13^C/^12^C. The isotopic fractionation factor *α*_*db*_ is defined by the isotopic ratio of CO_2_ divided by the isotopic ratio of HCO_3_^–^, i.e. $\alpha_{db}=R_{CO_{2}}/R_{HCO_{3}^{-}}$. Using the equilibrium fractionation (*ε*_*db*_), the fractionation factor between CO_2_ and HCO_3_^-^ can be calculated for the external medium as well as for the cytosol according to:

$$\alpha_{db,\;ext}=1+\varepsilon_{db}/1000$$ (6)

$$\alpha_{db,\;cyt}=1+\varepsilon_{cyt}/1000$$ (7)

While the equilibrium value *ε*_*db*_ is –9‰ (i.e. CO_2_ is isotopically lighter than HCO_3_^–^; Mook *et al.*, 1974), *ε*_*cyt*_ can significantly deviate from this value due to kinetic effects. The uncatalysed conversion of HCO_3_^–^ to CO_2_ shows a kinetic fractionation of –22‰, whereas the formation of HCO_3_^–^ from CO_2_ is associated with a kinetic fractionation of +13‰ (Marlier and O’Leary, 1984). Hence, the actual value of *ε*_*cyt*_ is determined by the disequilibrium between CO_2_ and HCO_3_^–^ in the cytosol, which depends on all fluxes in and out of the cytosol, and on the internal CO_2_ and HCO_3_^–^ concentrations, which cannot be calculated in the framework of a flux-based model. Assuming a unidirectional conversion of CO_2_ to HCO_3_^–^ in the cytosol, a value of +13‰ for *ε*_*cyt*_ will be adopted. By setting *ε*_*cyt*_ to +30‰, a potential fractionation by the NDH-1_4_ complex will be taken into account. The situation where the conversion of CO_2_ to HCO_3_^–^ in the cytosol is not completely unidirectional will be considered by setting *ε*_*cyt*_ to +8‰.

The *R* associated with *F*_*fix*_ can be written in terms of the isotopic fractionation against ^13^C by RubisCO described by the factor *α*_*Rub*_ = *R*_*carb*_ / *R*_*POC*_, where *R*_*carb*_ is the isotopic ratio of CO_2_ in the carboxysome and *R*_*POC*_ is the isotopic ratio of POC. The value of *α*_*Rub*_ is calculated from the intrinsic RubisCO fractionation *ε*_*Rub*_ (assuming an intermediate value of +25‰; Roeske and O’Leary, 1984; Guy *et al.*, 1993):

$$\alpha_{Rub}=1+\varepsilon_{Rub}/1000$$ (8)

Given the isotopic ratios (*R*) of CO_2_ and the isotopic fractionation factors between HCO_3_^–^ and CO_2_ expressed as *α*_*bd*_ = 1/*α*_*db*_, the flux balance equations for ^13^C can be derived from eqns 4 and 5 for the cytosol and the carboxysome, respectively:

$$\begin{array}{l} R_{ext}F_{cyt,\;CO_{2}}+\alpha_{bd,\;ext}R_{ext}F_{cyt,\;HCO_{3}^{-}}+R_{carb}F_{carb,\;out}\\ -\;R_{cyt}F_{cyt,\;out}-\;R_{cyt}F_{carb,\;CO_{2}}-\;\alpha_{bd,\;cyt}R_{cyt}F_{carb,\;HCO_{3}^{-}}=0 \end{array}$$ (9)

$$\begin{array}{l} \alpha_{bd\text{,}\;cyt}R_{cyt}F_{carb,\;HCO_{3}^{-}}+R_{cyt}F_{carb,\;CO_{2}}\\ -\;R_{carb}F_{carb,\;out}-R_{carb}F_{fix}/\alpha_{Rub}=0\; \end{array}$$ (10)

*R*_*cyt*_ is the isotopic ratio of CO_2_ in the cytosol. The overall isotopic fractionation by the cell is defined with respect to the isotopic composition of CO_2_ in the external medium (*R*_*ext*_):

$$\varepsilon_{p}=\left(\frac{R_{ext}}{R_{POC}}-1\right)\;\times \;1000=\left(\alpha_{Rub}\frac{R_{ext}}{R_{carb}}-1\right)\;\times \;1000$$ (11)

The ratio *R*_*ext*_/*R*_*carb*_ reflects the impact of the inner compartment on the isotopic fractionation and can be calculated from flux balance eqns 9 and 10. Eqn 10 can be solved for *R*_*cyt*_, which in turn is substituted into eqn 9, yielding the ratio:

$$\begin{array}{l} \frac{R_{ext}}{R_{carb}}=\frac{\begin{array}{l} \left(F_{fix}/\alpha_{Rub}+F_{carb,\;out}\right)\left(F_{carb,CO_{2}}+\alpha_{bd,\;cyt}F_{carb,HCO_{3}^{-}}+F_{cyt,\;out}\right)\\ {}_{} \end{array}}{\left(F_{cyt,\;CO_{2}}+\alpha_{bd,\;ext}F_{cyt,\;HCO_{3}^{-}}\right){\left(F_{carb,\;CO_{2}}+\alpha_{bd,\;cyt}F_{carb,\;HCO_{3}^{-}}\right)}^{{}^{}}}-\frac{F_{carb,\;out}}{\left(F_{cyt,\;CO_{2}}+\alpha_{bd,\;ext}F_{cyt,\;HCO_{3}^{-}}\right)}\\ \;=\frac{F_{cyt,\;out}}{\alpha_{Rub}\left(F_{cyt,\;CO_{2}}+\alpha_{bd,\;ext}F_{cyt,\;HCO_{3}^{-}}\right)}\times \;\left(\frac{F_{fix}}{F_{cyt,\;out}}+\frac{F_{fix}+\alpha_{Rub}F_{carb,\;\;out}}{F_{carb,\;CO_{2}}+\alpha_{bd,\;cyt}F_{carb,\;HCO_{3}^{-}}}\right)\; \end{array}$$ (12)

This solution is valid for arbitrary combinations of fluxes as long as the constraints imposed by flux balance equations 4 and 5 are obeyed:

$$\begin{array}{l} F_{fix}=F_{carb,\;CO_{2}}+F_{carb,\;HCO_{3}^{-}}-F_{carb,\;out}\\ \;=F_{cyt,\;CO_{2}}+F_{cyt,\;HCO_{3}^{-}}-F_{cyt,\;out}\; \end{array}$$ (13)

Given the fractional contribution of HCO_3_^–^ to total C_i_ uptake into the cytosol (*a*_*cyt*_) and the carboxysome (*a*_*carb*_), as well as the leakage out of the cytosol (*L*_*cyt*_) and the carboxysome (*L*_*carb*_), eqns 6 to 8 and 11 to 13 can be used to derive the overall isotopic fractionation:

$$\begin{array}{l} \varepsilon_{p}=\frac{a_{cyt}\varepsilon_{db}}{1-a_{cyt}\varepsilon_{db}/10^{3}}+L_{cyt}\frac{\left(a_{carb}\varepsilon_{\text{cyt}}+L_{carb}\varepsilon_{Rub}\right)}{\left(1-a_{cyt}\varepsilon_{db}/10^{3}\right)\left(1-a_{carb}\varepsilon_{cyt}/10^{3}\right)}\\ \;\approx a_{cyt}\varepsilon_{db}+L_{cyt}\left(a_{carb}\varepsilon_{cyt}+L_{carb}\varepsilon_{Rub}\right)\text{.} \end{array}$$ (14)

Solving the approximated solution for *L*_*cyt*_ yields the following:

$$L_{cyt}=\frac{\varepsilon_{p}-a_{cyt}\varepsilon_{db}}{a_{carb}\varepsilon_{cyt}+L_{carb}\varepsilon_{Rub}}$$ (15)

The approximate solution can be considered as a generalization of the original function given by Sharkey and Berry (1985), accounting for two compartments. The authors assumed that the cell takes up HCO_3_^–^ into a single compartment and subsequently converts it to CO_2_; hence there is no HCO_3_^–^ inside the cell. The compatibility of our model with the original function can be confirmed by comparing *ε*_*p*_ for *L*_*carb*_ = 1 (i.e. no second compartment) and *a*_*carb*_ = 0 (i.e. only CO_2_ uptake into the carboxysome).

As pointed out by Schulz *et al.* (2007), diffusive CO_2_ fluxes generally need to be added to cellular fluxes measured by MIMS (Badger *et al.*, 1994) when relating them to ^13^C fractionation. For membrane permeability exceeding 10^–4^ cm s^–1^, as proposed for a diatom (~10^–2^ cm s^–1^; Hopkinson *et al.*, 2011), diffusive CO_2_ fluxes are high and internal CO_2_ concentrations approach those of the cell’s exterior (Supplementary Figure S2). In this case, gross CO_2_ efflux estimated by MIMS would be underestimated, which could explain part of the discrepancy between MIMS-based leakage and estimates based on eqn 4 (Sharkey and Berry, 1985). While there is, to our knowledge, no recent data on the membrane permeability of cyanobacteria available, older studies on cyanobacteria state significantly lower values, approaching 10^–5^ cm s^–1^ (Badger *et al.*, 1985; Marcus *et al.*, 1986), which are in line with diffusive CO_2_ fluxes being low enough to allow for considerable CO_2_ accumulation in the cell (Supplementary Figure S2). Using this permeability, the effect of diffusive CO_2_ influx on leakage obtained by our model was estimated, yielding maximum changes in the order of a few percent, which were thus neglected. In view of the uncertainties in this parameter, measurements of membrane permeability of cyanobacteria are needed to improve future estimates of internal C fluxes.

#### Internal C_i_ fluxes and fractionation—model application

**Fig. 4.**
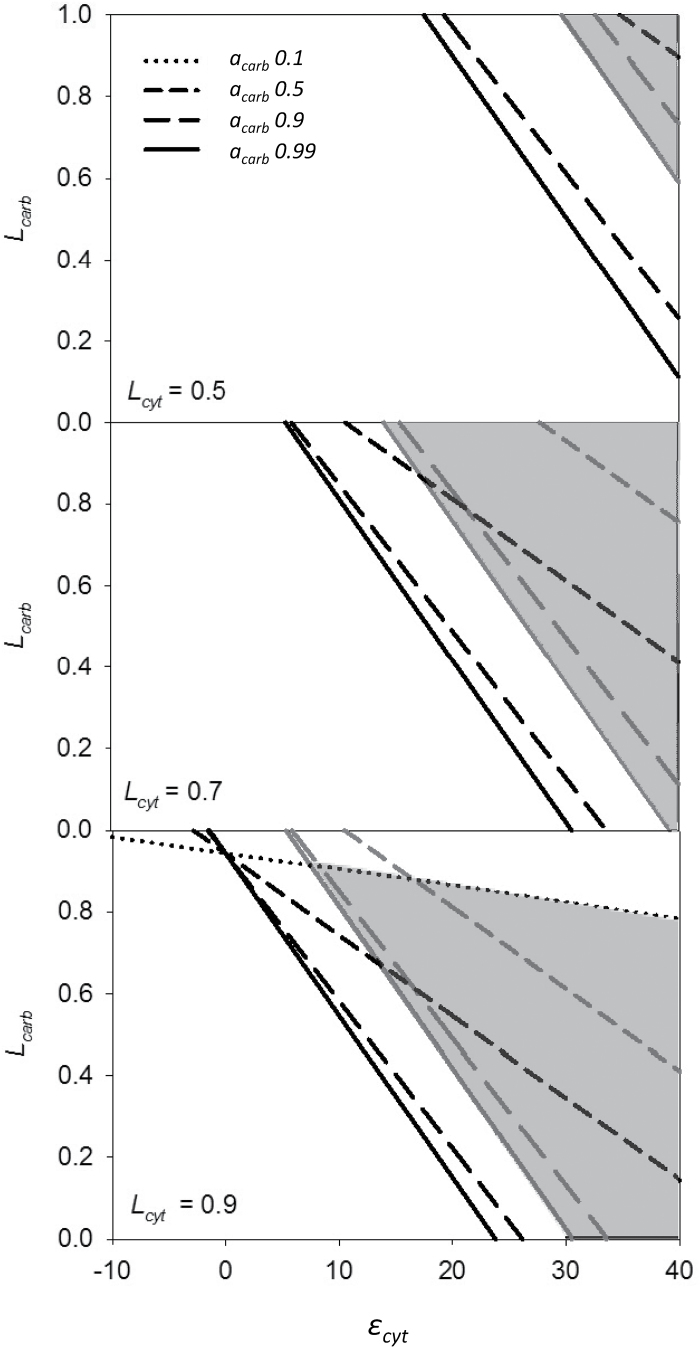
Interrelationship between *L*_*carb*_, *ε*_*cyt*_, and *a*_*carb*_ in the model, depicted for different values of *L*_*cyt*_ and *ε*_*p*_. The shaded areas mark the range of possible values for *L*_*carb*_ and *ε*_*cyt*_ that could reconcile our measurements of isotopic composition with measured external C_i_ fluxes. Black and grey lines are based on *ε*_*p*_ measured in cells acclimated to 380 and 1400 µatm *p*CO_2_, respectively.

To test the sensitivity of our model, the potential effect of changes in *a*_*cyt*_ on *ε*_*p*_ was quantified, using the maximum variability observed in our study (0.84 vs 0.76) while leaving all other parameters constant. This variability can explain a change in *ε*_*p*_ by not more than 0.7‰. Thus, *a*_*cyt*_ can be excluded as a main driver behind the variability in *ε*_*p*_ (or leakage estimates), even if variability in *a*_*cyt*_ is severely underestimated. Applying the model to our measured fluxes and *ε*_*p*_ values, a range of different possible scenarios for intracellular fluxes and fractionation in the cytosol is obtained (Fig. 4).

According to these interrelations, while at *L*_*cyt*_ according to our MIMS measurements (0.5), only a very high fractionation in the cytosol (*ε*_*cyt*_) can explain our results, at *L*_*cyt*_ ≥ 0.7, there is a large range of possible combinations of parameters (see shaded areas in Fig. 4). As we aim to find parameters that can explain *ε*_*p*_ in both of our *p*CO_2_ treatments, the high *ε*_*p*_ measured in cells grown at 1400 µatm constrains the range of possible values, while ε_*p*_ of cells grown at 380 µatm could be explained by a larger range of values for *a*_*carb*_ and *L*_*carb*_ (Fig. 4). High values for *a*_*carb*_ and *L*_*carb*_ (both approaching 1) allow for a larger range of possible values of *ε*_*cyt*_ to explain our measured *ε*_*p*_ (Fig. 4). Due to the high contribution of HCO_3_^–^ to C_i_ uptake and the additional conversion of CO_2_ to HCO_3_^–^ by the NDH complex, *a*_*carb*_ is likely to be close to 1, most probably exceeding *a*_*cyt*_ measured in our experiment (0.82). Moreover, high diffusive CO_2_ influx into the carboxysome seems unlikely in view of the supposed function of the carboxysome as a diffusion barrier to CO_2_ (e.g. Reinhold *et al.*, 1989). While comparison experiments with CA knockout mutants with intact and broken carboxysomes confirmed that the carboxysome shell impedes diffusion of CO_2_ (Dou *et al.*, 2008), the pores in the hexamer protein subunits of the shell are supposed to be permeable to small, negatively charged molecules such as HCO_3_^–^ (Tsai *et al.*, 2007; Klein *et al.*, 2009; Espie and Kimber, 2011). Despite the low CO_2_ permeability, high rates of CO_2_ efflux, and thus high *L*_*carb*_, are likely due to the very high accumulation factor (two to three orders of magnitude; this study and Kaplan *et al.*, 1980). A value for *L*_*carb*_ of 0.9 is therefore used in the model scenarios described in the following (Table 2). Using eqn 13, the following expression for the ratio of internal to external C_i_ fluxes can be derived:

**Table 2.** Different scenarios of external and internal C_i_ fluxes that can reconcile measurements of C_i_ fluxes by MIMS and *ε*_*p*_ values obtained in this study (scenarios 1, 2 and 4 to 6) and by Kranz *et al.* (2010, scenario 3)^a^

Scenario	*p*CO_2_	*ε*_*p*_	*a*_*cyt*_	*L*_*MIMS*_	*L*_*13C*_	*L*_*cyt*_	*L*_*carb*_	*a*_*carb*_	*ε*_*cyt*_
		Measured				Modelled			
1	1400	20	0.8	0.5	1.1	0.8	0.9	1	13
2	380	14	0.8	0.5	0.8	0.6	0.9	1	13
3	180	7	0.8	0.4	0.6	0.4	0.9	1	13
4	1400	20	0.8	0.5	1.1	0.9	0.9	1	8
5	1400	20	0.8	0.5	1.1	0.5	0.9	1	30
6	380	14	0.8	0.5	1.1	0.6	0.9	0.7	20

^a^ *ε*_*p*_, *a*_*cyt*,_ and *L*_*MIMS*_ were measured; *L*_*13C*_ was calculated from *ε*_*p*_ according to Sharkey and Berry (1985); remaining values are model input parameters and model results (*L*_*cyt*_ *, L*_*carb*_, *a*_*carb*_, and *ε*_*cyt*_).

$$\frac{F_{carb,CO_{2}}+F_{carb,HCO_{3}^{-}}}{F_{cyt,CO_{2}}+F_{cyt,HCO_{3}^{-}}}=\frac{1-{\text{L}}_{cyt}}{1-{\text{L}}_{carb}}$$ (16)

For the chosen value for *L*_*carb*_ of 0.9 and the measured *L*_*cyt*_ of 0.5, eqn 16 yields a ratio of internal vs external C_i_ cycling of 5.

Compared to estimates based on Sharkey and Berry (1985), our model significantly improved the compatibility of leakage estimates with those obtained by MIMS measurements (Table 2). The maximum fractionation that could be achieved in an uncatalysed reaction from CO_2_ to HCO_3_^–^ is +13‰ (O’Leary *et al.*, 1992). With this kinetic fractionation, *ε*_*p*_ values measured for the two *p*CO_2_ levels can be explained by leakage values from the cytosol (*L*_*cyt*_) of 0.8 and 0.6, respectively (scenarios 1 and 2, Table 2), which are significantly lower than the estimates based on the function by Sharkey and Berry (*L*_*13C*_ = 1.1 and 0.8, respectively). The remaining difference to leakage estimates obtained by MIMS (*L*_*MIMS*_ = 0.5) could be explained by an underestimation of leakage by the MIMS approach, as discussed above. Assuming that the conversion between CO_2_ and HCO_3_^–^ in the cytosol was not completely unidirectional, *ε*_*cyt*_ could range between +13‰ and –9‰ (equilibrium fractionation; O’Leary *et al.*, 1992). To simulate this intermediate scenario, an *ε*_*cyt*_ of +8‰ is assumed (senario 4, Table 2), yielding an *L*_*cyt*_ of 0.9 for the high CO_2_ treatment.

Kinetic fractionation could be achieved by the NDH complex or by creation of a strong disequilibrium in the cytosol, minimizing the back-reaction from HCO_3_^–^ to CO_2_. Mutants of *Synechococcus* expressing human CA in the cytosol were unable to accumulate C_i_ (Price and Badger, 1989b), suggesting that HCO_3_^–^ is accumulated in the cytosol, and that a chemical disequilibrium in the cytosol favours the reaction from HCO_3_^–^ to CO_2_ rather than the opposite direction. This strongly argues for a fractionating enzyme instead of a purely chemical disequilibrium driving unidirectional CO_2_ to HCO_3_^–^ conversion in the cytosol. Assuming that NDH not only drives the unidirectional conversion of CO_2_ to HCO_3_^–^ but also discriminates against ^13^C during the reaction, leakage estimates by our model can be further reconciled with MIMS-based estimates. In a scenario assuming an upper estimate for *ε*_*cyt*_ of +30‰ (scenario 5, Table 2), which is within the range of fractionation measured in other enzymes such as RubisCO, our MIMS-measured data can be reproduced even for the high *p*CO_2_ treatment (*L*_*MIMS*_ = *L*_*cyt*_ = 0.5; Table 2). Note that in combination with one of the other factors discussed above, such as an underestimation of *L*_*carb*_ or of MIMS-based leakage estimates, enzymatic fractionation less than +30‰ could also explain our measurements (cf. e.g. scenarios with *ε*_*cyt*_ of 13‰; Table 2). Although CA and NDH have been proposed to have similar reaction mechanisms (Price *et al.*, 2002), our model results suggest that the fractionation by the NDH complex is different from that of CA (1‰ for conversion of CO_2_ to HCO_3_^–^; Paneth and O’Leary, 1985). This might be due to the fact that the subunit carrying out the hydration reaction in the NDH-1_4_ complex (chpX) is embedded in a larger functional unit including the transmembrane proton channel and is associated with the electron transport chain. To confirm the differences in fractionation between these enzyme complexes, however, further work using experimental approaches would be necessary, e.g. by comparing cyanobacterial mutant strains.

Since our MIMS measurements showed that leakage was unaffected by *p*CO_2_ and the slight changes in *a*_*cyt*_ could not explain the observed variation in *ε*_*p*_, *a*_*carb*_ and/or *ε*_*cyt*_ would need to vary with *p*CO_2_ to explain the observed CO_2_-dependence of *ε*_*p*_. An increase in the activity of the NDH complex could yield an increase in *a*_*carb*_ as well as *ε*_*cyt*_ and therewith *ε*_*p*_. With an increase in *a*_*carb*_ from 0.7 to 1 and an increase in *ε*_*cyt*_ from 20 to 30‰, the measured increase in *ε*_*p*_ between 380 and 1400 µatm can be explained, almost reproducing MIMS-measured leakage in both scenarios (scenarios 5 and 6, Table 2). A higher activity of NDH at high *p*CO_2_ may seem unexpected in view of its supposed role as part of the CCM. However, in contrast to other components of the CCM such as HCO_3_^–^ transporters, the reaction catalysed by the NDH complex contributes to ATP regeneration rather than consuming energy, and thus a downregulation of NDH at high *p*CO_2_ would not provide any energetic benefit to the cell. A positive correlation with *p*CO_2_ might be coupled to the small CO_2_ effect on *a*_*cyt*_ (Table 1; Supplementary Figure 1C), increasing the activity of the NDH complex at high *p*CO_2_ levels in the acclimations in response to a higher availability of its substrate CO_2_. Note that the change in *a*_*cyt*_ may have been underestimated in our study due to the constant pH in MIMS measurements. Due to its function as a proton pump in the thylakoid membrane, activity of the NDH complex can increase the ratio of ATP to NADPH available in the cell. The production of ATP by high NDH activity at high *p*CO_2_ could, in turn, contribute to the increased ATP requirement to fuel N_2_ fixation in *Trichodesmium* under ocean acidification (e.g. Kranz *et al.*, 2010; Eichner *et al.*, 2014).

Kranz *et al.* (2010) compared leakage estimates based on MIMS and ^13^C for *Trichodesmium* grown under different *p*CO_2_ levels as well as light intensities. Applying our model to this data set, MIMS-based leakage estimates could be reproduced with unidirectional fractionation for a low *p*CO_2_ treatment with light intensities similar to our experiment (200 µmol photons m^–2^ s^–1^; scenario 3, Table 2). In high *p*CO_2_ and low light treatments, the difference between *L*_*MIMS*_ and *L*_*cyt*_ was larger and could consequently only be reconciled with *ε*_*cyt*_ values larger than +30‰. Short-term exposure to high light intensities (300 µmol photons m^–2^ s^–1^) in our experiment affected CO_2_ efflux in cells acclimated to high *p*CO_2_ (ANOVA, *P* < 0.05; data not shown). Such light sensitivity generally suggests CO_2_ efflux to be closely associated to photosynthetic electron transport. As this light effect was only observed under high *p*CO_2_, i.e. conditions associated with higher NDH activity according to our model results, the interrelation of CO_2_ efflux and the NDH complex is further corroborated. The observed light effects on CO_2_ efflux (this study) and *ε*_*p*_ (Kranz *et al.*, 2010) impose interesting questions with regard to a potential regulation of the NDH complex by the electron transport chain (redox state and/or proton gradient), which should be investigated in future studies. A better understanding of the regulation of the NDH complex is essential to improve confidence in explaining the effects of *p*CO_2_ as well as light on internal C_i_ fluxes and potential feedbacks on cellular energy budgets of this key N_2_ fixer.

### Conclusion and outlook

This study demonstrates that internal C_i_ fluxes via the NDH-1_4_ complex need to be considered not only in terms of cellular C_i_ acquisition, but also with regard to ^13^C fractionation and cellular energy status. The comparison of direct measurements of C_i_ fluxes with estimates based on isotopic composition revealed that ^13^C fractionation in *Trichodesmium* cannot be adequately described by only considering external C_i_ fluxes.

Compatibility with direct leakage measurements was improved by applying a model accounting for internal C_i_ fluxes around the carboxysome, e.g. via the NDH complex, providing a generalization of the model of Sharkey and Berry (1985) applicable for two compartments. In these model calculations, a large fraction of HCO_3_^–^ uptake into the carboxysome (0.7–1, Table 2) and high leakage from the carboxysome (>0.9, Table 2) are assumed, in accordance with the current understanding of carboxysome functioning. A large range of fractionation values for the cytoplasm, representing uncatalysed, unidirectional fractionation as well as enzymatic fractionation by the NDH complex, could significantly improve compatibility of leakage estimates (Table 2), even though the exact interplay of these processes still has to be resolved. While the lack of recent literature data on membrane permeability of cyanobacteria clearly demands future measurements, our model calculations are insensitive to this parameter within a certain range of permeability estimates (<10^–4^ m^–2^ s^–1^). Similarly, the model is independent of other potentially uncertain assumptions such as permeability of the carboxysome and pH values for the different compartments. Once future studies improve confidence in these parameters, a kinetic model can be used to predict internal concentrations and individual C_i_ fluxes. The agreement of model results with measured values could further be improved by accounting for possible ^13^C enrichment of CO_2_ leaking out of the cell as well as a disequilibrium situation in the surroundings of the cell (altering the isotopic signature of HCO_3_^–^ taken up).

The model applied here could also be used to improve estimates of leakage based on ^13^C signatures for other species in which several compartments and/or internal C_i_ fluxes play an important role. For phytoplankton groups that are relevant in terms of paleo-proxies, this could have important implications for the interpretation of C isotope signals.

## Supplementary material

Supplementary Figure S1. DIC-saturated rates of C fixa tion (V_max_), half saturation DIC concentrations (K_1/2_) and HCO_3_^–^ : C_i_ uptake ratios measured at three time points during the day in *Trichodesmium* grown under two *p*CO_2_ levels and N sources (N_2_ and NO_3_^–^).

Supplementary Figure S2. The dependence of intracellular CO_2_ concentrations on membrane permeability.

## Funding

This work was supported by the European Research Council under the European Community’s Seventh Framework Programme (FP7/2007–2013), ERC grant agreement (205150), and the US National Science Foundation (EF 1040965).

## Supplementary Material

Supplementary Data

## Abbreviations:

*a*_*carb*_: fractional contribution of HCO_3_^–^ to total C_i_ uptake into the carboxysome
*acyt*: fractional contribution of HCO_3_^–^ to total C_i_ uptake into the cytosol
CA: carbonic anhydrase
CCM: carbon-concentrating mechanism
chl *a*: chlorophyll *a*
Ci: inorganic carbon
DIC: dissolved inorganic carbon
K_1/2_: half-saturation concentration
*L*_13C_: leakage calculated from ^13^C fractionation
*L*_carb_: modelled leakage from the carboxysome
*L*_cyt_: modelled leakage over the plasma membrane
*L*_*MIMS*_: leakage estimated by MIMS
MIMS: membrane inlet mass spectrometry
POC: particulate organic carbon
PQ: photosynthetic quotient
V_max_: maximal rate
*ε*_*cyt*_: ^13^C fractionation in the cytosol
*ε*_*db*_: ^13^C equilibrium fractionation in the external medium
*ε*_*p*_: total ^13^C fractionation during POC formation
*ε*_*Rub*_: 13C fractionation by RubisCO.
