# Supplementary material for: Cellular inorganic carbon fluxes in Trichodesmium: a combined approach using measurements and modelling
Source: J Exp Bot. 2014 Nov 26;66(3):749–59. doi: 10.1093/jxb/eru427 (PMC4321539; doi:10.1093/jxb/eru427)
Supplement: Supplementary Data [file supp_66_3_749__index.html]

Cellular inorganic carbon fluxes in Trichodesmium: a combined approach using measurements and modelling — Cellular inorganic carbon fluxes in Trichodesmium: a combined approach using measurements and modelling — Supplementary Data 

# Cellular inorganic carbon fluxes in *Trichodesmium*: a combined approach using measurements and modelling

## Supplementary Data

Data files

**Files in this Data Supplement:**

- Supplementary Data - Supplementary Data
