# Supplementary material for: Cellular inorganic carbon fluxes in Trichodesmium: a combined approach using measurements and modelling
Source: J Exp Bot. 2014 Nov 26;66(3):749–59. doi: 10.1093/jxb/eru427 (PMC4321539; doi:10.1093/jxb/eru427)
Supplement: Supplementary Data [file supp_eru427_jexbot129304_file001.pdf]

Cellular inorganic carbon fluxes in *Trichodesmium*: A combined approach of measurements and modeling

Meri Eichner, Silke Thoms, Sven A. Kranz, Björn Rost

SUPPLEMENTARY DATA

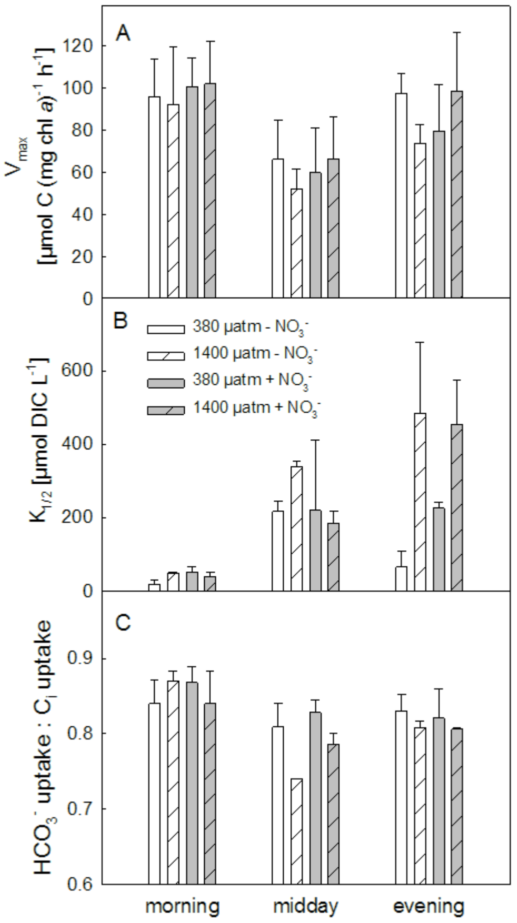

Supplementary Fig. S1 A&B: DIC-saturated rates of C fixation (V_{max}) and half saturation DIC concentration ($K_{1/2}$) measured at three time points during the day in *Trichodesmium* grown under two pCO_2 levels and N sources (N_2 and NO_3^-). Error bars denote 1 SD ($n \geq 3$; except for 1400 µatm –NO_3^- morning and midday and 380 µatm + NO_3^- morning with n = 2). C: HCO_3^- : C_i uptake (calculated from the respective V_{max} values). Error bars denote 1 SD ($n \geq 3$; except for 1400 µatm –NO_3^- morning (n = 2) and midday (n = 1)).

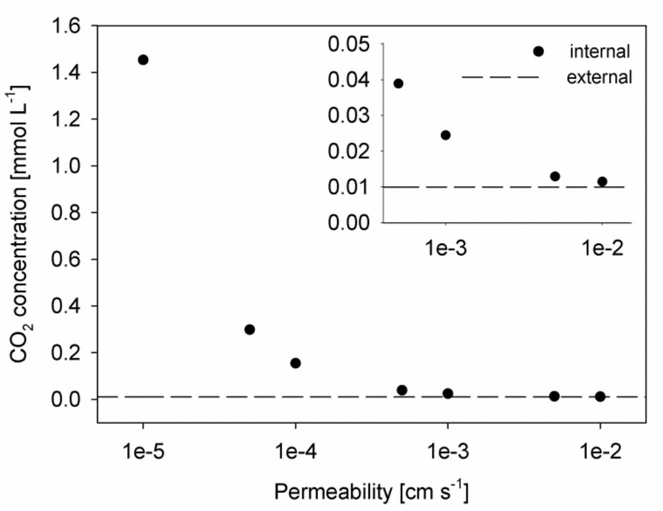

Supplementary Fig. S2: Dependence of intracellular CO_2 concentrations on the membrane permeability. While at permeability of ~ $1x10^{-5}$, internal concentrations exceed the external concentrations by several orders of magnitude, at permeability > $1x10^{-4}$ internal concentrations approach external concentrations. The inset shows a magnification for the high permeability range. Internal CO_2 concentrations were derived using the following equation:

$$L_{MIMS} = \frac{P \cdot A \cdot \Delta[CO_2]}{C_{fix} + P \cdot A \cdot \Delta[CO_2]}$$

where L_{MIMS} is leakage measured by MIMS (using a typical value of 0.5), P is the membrane permeability, A is the surface area of the cell (using a typical value of $1.5x10^{-6}$ cm^2), $\Delta[CO_2]$ is the difference between internal and external CO_2 concentration and C_{fix} is the carbon fixation rate measured by MIMS (using a typical value of $8x10^{-14}$ mol $cell^{-1}$ h^{-1}).
